# Illuminating the Genomic Landscape of *Lactiplantibacillus plantarum* PU3—A Novel Probiotic Strain Isolated from Human Breast Milk, Explored through Nanopore Sequencing

**DOI:** 10.3390/microorganisms11102440

**Published:** 2023-09-28

**Authors:** Daniela Mollova, Mariyana Gozmanova, Elena Apostolova, Galina Yahubyan, Ilia Iliev, Vesselin Baev

**Affiliations:** 1 Faculty of Biology, Department of Biochemistry and Microbiology, University of Plovdiv, Tzar Assen 24, 4000 Plovdiv, Bulgaria; dmollova.bio@uni-plovdiv.bg (D.M.); iliailiev@uni-plovdiv.bg (I.I.); 2 Faculty of Biology, Department of Plant Physiology and Molecular Biology, University of Plovdiv, Tzar Assen 24, 4000 Plovdiv, Bulgaria; mariank@uni-plovdiv.bg (M.G.); eapostolova@uni-plovdiv.bg (E.A.); gyahubyan@uni-plovdiv.bg (G.Y.)

**Keywords:** *Lactiplantibacillus plantarum*, LAB, genome sequencing, ONT, probiotics

## Abstract

*Lactiplantibacillus plantarum* stands out as a remarkably diverse species of lactic acid bacteria, occupying a myriad of ecological niches. Particularly noteworthy is its presence in human breast milk, which can serve as a reservoir of probiotic bacteria, contributing significantly to the establishment and constitution of infant gut microbiota. In light of this, our study attempted to conduct an initial investigation encompassing both genomic and phenotypic aspects of the *L. plantarum* PU3 strain, that holds potential as a probiotic agent. By employing the cutting-edge third-generation Nanopore sequencing technology, *L. plantarum* PU3 revealed a circular chromosome of 3,180,940 bp and nine plasmids of various lengths. The *L. plantarum* PU3 genome has a total of 2962 protein-coding and non-coding genes. Our in-depth investigations revealed more than 150 probiotic gene markers that unfold the genetic determinants for acid tolerance, bile resistance, adhesion, and oxidative and osmotic stress. The in vivo analysis showed the strain’s proficiency in utilizing various carbohydrates as growth substrates, complementing the in silico analysis of the genes involved in metabolic pathways. Notably, the strain demonstrated a pronounced affinity for D-sorbitol, D-mannitol, and D-Gluconic acid, among other carbohydrate sources. The in vitro experimental verification of acid, osmotic and bile tolerance validated the robustness of the strain in challenging environments. Encouragingly, no virulence factors were detected in the genome of PU3, suggesting its safety profile. In search of beneficial properties, we found potential bacteriocin biosynthesis clusters, suggesting its capability for antimicrobial activity. The characteristics exhibited by *L. plantarum* PU3 pave the way for promising strain potential, warranting further investigations to unlock its full capacity and contributions to probiotic and therapeutic avenues.

## 1. Introduction

The term “microbiome” refers to the collection of microorganisms present in a specific ecological location or habitat. In the human body, these microorganisms can be commensal, symbiotic, or pathogenic, constituting the human microbiota. In the past few years, advancements in molecular techniques have made it increasingly effortless to detect and identify the ensemble of bacterial species present in the human body [1]. Advancements in bacterial detection methods, particularly those that are not reliant on traditional culture techniques (like 16S rRNA sequencing and WGS approaches), have revealed significantly greater bacterial diversity in breast milk than initially anticipated.

Human breast milk is rich in carbohydrates, essential fatty acids, proteins, vitamins, and minerals, making it a crucial and highly valued source of nourishment for babies. As a result, it is widely acknowledged as the benchmark for infant nutrition [2]. Additionally, it contains various beneficial components such as lactoferrin, immune cells, regulatory cytokines, and other biologically active substances. These elements collectively create a favorable environment in the gut of newborns, promoting the colonization of beneficial bacteria [3]. The development and composition of the human gut microbiota during the initial 1000 days of life have a crucial impact on the host’s overall health and well-being in the future, making it an indispensable factor [4]. Based on our understanding, it is widely accepted that breast milk may contain microbes originating from two main sources: maternal gut translocation (through the entero-mammary pathway) and exposure to environmental bacteria while breastfeeding [5]. It has also been demonstrated to act as a probiotic bacterial reservoir for the baby’s intestine, containing beneficial microorganisms like *Staphylococcus*, *Streptococcus*, *Bifidobacteria*, and lactic acid bacteria (LAB). LAB such as *L. gasseri*, *L. salivarius*, *L. rhamnosus*, *L. plantarum*, and *L. fermentum* are found in breast milk and are considered probiotic species [6].

*Lactiplantibacillus plantarum* (previously known as *Lactobacillus plantarum*), a type of LAB with a positive Gram stain, demonstrates both ecological and metabolic flexibility. It can flourish in various environments, such as fermented foods, meats, and plants [7]. It is commonly believed that different bacterial strains adapt to specific environments by undergoing genome specialization. This process involves the decay of unused genes and the enrichment of genes that provide fitness advantages in their particular habitat. As a result, bacterial strains from the same ecological niche tend to have similar genetic signatures, ensuring their adaptation to a specific environment [8].

*L. plantarum* boasts one of the largest genomes known among LAB [9], enabling it to thrive in various environmental conditions, including the gastrointestinal tract, where it readily colonizes the intestines of humans and other mammals [10,11]. Moreover, numerous strains of this species have demonstrated advantageous effects on the host, such as their ability to positively regulate the immune system [12]. As a result of these characteristics, *L. plantarum* is highly regarded as a versatile bacterial strain extensively utilized in the food industry, serving as both a starter culture and a probiotic [13]. The successful use of probiotics relies on several factors, with the species, strain, and composition of the probiotic product being crucial. To be an effective probiotic, a bacterial species must possess specific functional properties. The primary characteristics include the ability to withstand gastric acid and bile salts, adhere to the intestinal mucosa, and remain unaffected by antibiotics. Furthermore, these beneficial microbes positively influence the balance of intestinal microflora, enhance intestinal integrity and mobility, regulate host immune responses, and demonstrate antimicrobial or competitive activity against potentially harmful bacteria [14].

With the rapid advancements in next-generation sequencing techniques, multiple strains of *L. plantarum* have undergone complete genome sequencing. So far, there are more than 200 fully completed strains in the NCBI database. Recently, this scientific–technological progress has significantly contributed to our comprehension of the associations between strains and functions [15,16].

Oxford Nanopore Technologies (ONT) is a novel state-of-the-art single-molecule-based sequencing platform (third-generation sequencing) that offers the distinct advantage of real-time sequencing and portability, making it an ideal and accessible technology even for smaller labs [17,18]. One of the key strengths of the ONT nanopore sequencer lies in its ability to generate exceptionally long reads without any limitations on read length. This feature proves highly beneficial in resolving complex structural and repetitive regions present in bacterial genomes [19]. With such output, the bacterial strains’ genome reconstruction and de novo assembly can be performed far more accurately and efficiently compared to other sequencing platforms [20], usually resulting in complete circular closed molecules. Moreover, with sufficient coverage (>x30), this technology can produce high-quality genomes of strains even without hybrid assembly using NGS data, which considerably lowers the cost [21,22,23].

This study focuses on the *L. plantarum* strain PU3 isolated from human breast milk, for which complete the genome have been sequenced using Nanopore MinION. The objective is to conduct comprehensive genomic investigations to highlight important features and properties that can reveal the probiotic capacity of the strain. Special attention has been directed towards the identification of the genes potentially involved in stress responses (temperature, pH, bile, osmotic pressure, and oxidative stress) and those related to the strain adaptation in the host gastrointestinal tract. We found more than 150 genes that could be linked to probiotic activity. Furthermore, some of these probiotic features, such as bile resistance, osmotic stress, and acid tolerance, were also investigated in vivo, complementing the in silico data. Moreover, the PU3 strain presents a versatile carbohydrate utilization capability, which may contribute to broad adaptability in various environments with different carbohydrates [10,24,25,26].

## 2. Materials and Methods

### 2.1. Subjects and Sample Collection

Before participating in the study, all subjects provided informed consent for inclusion. The research adhered to the principles outlined in the Declaration of Helsinki, and the protocol received approval from the Ethics Committee of the University of Plovdiv (No 6/06.10.2022).

Breast milk samples were gathered from lactating mothers during the first six months following childbirth. The participants were healthy women who had given birth to full-term babies, either through vaginal delivery or C-section. To acquire the milk samples, the mothers were instructed to clean their breasts with water and then collect 15–20 mL of milk into a sterile container. These containers were subsequently kept at 4 °C until they were collected and transported to the laboratory. The milk samples underwent processing within 24 h of being donated.

### 2.2. Bacterial Isolation and Identification

Standard laboratory procedures were employed to collect and identify the bacterial samples. These methods included typical protocols for isolating bacteria from body fluids or anaerobic cultures. In order to isolate the anaerobic bacterial strains, the samples were plated on agar plates containing Lactobacillus de Man Rogosa and Sharpe (MRS) medium. Subsequently, the plates were kept in an anaerobic environment at 37 °C for 72 h. As a preliminary screening of Lactobacillus, we performed Gram staining and catalase tests. The isolated strains were preserved as stock cultures at −20 °C in MRS broth (Merck, Darmstadt, Germany) supplemented with 15% glycerol (Merck, Darmstadt, Germany) for subsequent analysis.

### 2.3. DNA Extraction, Sequencing, Assembly

Total DNA was extracted from isolate PU3 using the QIAamp DNA Microbiome Kit (QIAGEN, Hilden, Germany). DNA quantity and integrity were assessed using a Qubit 4 fluorometer (Thermo Fisher Scientific, Waltham, MA, USA) and agarose gel electrophoresis, respectively.

The long-read ONT library was prepared using a Ligation Sequencing Kit SQK-LSK109 (Oxford Nanopore Technologies, Oxford, UK) with 1 μg of total DNA, according to the manufacturer’s protocol and sequenced on a MinION using an R9.4.1 flow cell (Oxford Nanopore Technologies, Oxford, UK). Base calling, and quality control were performed offline using Guppy v6.5.7 (Oxford Nanopore Technologies, Oxford, UK). Adapter trimming was carried out using Porechop v.0.2.4 with default parameters (https://github.com/rrwick/Porechop, accessed on 25 September 2023).

De novo assembly was performed with Flye v2.9.2 under default parameters, excluding reads shorter than 1000 bp [27]. Assembly polishing was accomplished with the Racon v1.4.21 (https://github.com/isovic/racon, accessed on 25 September 2023) and Medaka v1.8.1 (https://github.com/nanoporetech/medaka, accessed on 25 September 2023) tools. The quality of the assembled sequence was assessed with the CheckM v1.1.6 tool [28,29]. A circular genome map was visualized from the single circular chromosome contig using the Proksee tool (https://proksee.ca/, accessed on 25 September 2023). MOB-Typer suite was used to assess the plasmid contigs.

### 2.4. Genome-Based Identification and MultiLocus Sequence Typing (MLST)

To identify the bacterial species, the average nucleotide identity (ANI) of the PU3 isolate was calculated with FastANI [30]. The genome was also used for MLST at PubMLST at https://pubmlst.orghttps://tygs.dsmz.de/, accessed on 25 September 2023 [31]. The Type (Strain) Genome Server (TYGS) was also used to create a whole-genome sequence-based phylogenetic tree (https://tygs.dsmz.de/, accessed on 25 September 2023) [32].

### 2.5. Genome Annotation

The assembly of the PU3 strain was submitted in NCBI Genomes for initial annotation using the Prokaryotic Genome Annotation Pipeline (PGAP) and accession number assignment [33]. Subsequently, the resulting GenBank file was used for further genome annotation using the Rapid Annotations using Subsystems Technology (RAST) web-server [34,35]. In addition, the functional annotations were carried out with the KEGG database and BlastKOALA tool using the predicted protein sequencing from the PGAP GenBank file [36]. Manual curation of the genes related to probiotic properties was conducted using RAST- and KEGG-derived annotations. Carbohydrate-active enzymes (CAZymes) within the PU3 genome were identified using the cbCAN3 tool and CAZy database (https://bcb.unl.edu/dbCAN2/, accessed on 25 September 2023).

The PU3 genome was searched for antimicrobial resistance (AMR) and virulence genes (VF) using the Abricate tool (https://github.com/tseemann/abricate, with default parameters, accessed on 25 September 2023) against the Comprehensive Antibiotic Resistance Database (CARD) [37], MEGARes DB [38], and Virulence Factor of Bacterial Pathogen database (VFDB) [39]. The AMR annotation was also enriched with BlastKOALA tool entries. The prediction of bacteriocin-related genes in the genome was fulfilled using the BAGEL4 webserver (http://bagel4.molgenrug.nl/, accessed on 25 September 2023) [40]. Clustered Regularly Interspaced Short Palindromic Repeats (CRISPR) and CRISPR-associated genes (Cas) were predicted with the CRISPRCasFinder tool using default parameters [41].

### 2.6. Metabolic Modeling

We used annotated proteins from the PGAP GenBank file to feed into the ModelSEED database for the generation of the draft metabolic model (https://modelseed.org/, accessed on 25 September 2023) [42]. The Gram-positive template was used. The resulting model was gapfilled manually for some substrates. The model was used for flux balance analysis (FBA) prediction of carbohydrate utilization and validation from the phenotype data. The pathways and reactions were visualized in MetExplore tool, which was input with the SBML model file from ModelSEED [43].

### 2.7. Assimilation of Different Types of Carbon Sources and Osmotic Sensitivity

We utilized Biolog’s Phenotype MicroArrays™ (PM) in conjunction with the Omnilog™ (USA) instrument to perform phenotypic screening. During the experiment, we incubated the samples, and the Omnilog instrument continuously read the plaques at 20 min intervals over a period of 24 h.

Data collection involved a two-step approach. First, Omnilog was used to measure the Optical Density (OD) in each well and directly assess cell proliferation. A redox-sensitive dye was used to measure the color change in each well based on NADH production, allowing the determination of metabolic activity. We were able to gather both types of measurements simultaneously, which enabled us to explore and understand the differences between metabolism and cell growth in terms of their phenotypic differences. A Gen III plate with inoculation fluid A was used for our experiments.

### 2.8. Bile Salt Tolerance

In this study, we investigated the ability of the tested strain to withstand different concentrations of bile salts. For this purpose, we used three different concentrations of bile salts: 0.3%, 1%, and 3%. They were added to the MRS medium, and the strain was cultured for 3 h. The optical density was measured at 0, 1, and 3 h after inoculation at a wavelength of 600 nm using the Beckman Coulter DU 730 instrument from California, USA, and the level of bacterial growth was recorded [44].

### 2.9. Acid Tolerance

The capacity to endure acidic conditions was evaluated by exposing samples to pH 3, pH 5, pH 7, pH 9 and pH 10 in MRS broth at 37 °C. To assess this, the spectrophotometric measurements were taken at a wavelength of 600 nm using the Beckman Coulter DU 730 instrument from California, USA. The readings were recorded at 0, 1, and 3 h of incubation, and this process was duplicated twice for each sample [45].

### 2.10. Determination of Antibiotic Sensitivity of Strain

Sharma et al.’s method [46] was used to evaluate the antibiotic sensitivity of the strains. The susceptibility to ten commonly used clinical antibiotics (Erythromycin, Amikacin, Gentamicin, Kanamycin, Amoxicillin, Ampicillin, Penicillin, Ciproflaoxacin, Chloramphenicol, and Ceftriaxone) was determined using the disc diffusion method. Antibiotics were obtained from Oxoid (Thermo Fisher Scientific, Wesel, Germany). Active cultures were prepared by adjusting the suspension densities to McFarland 0.5. Then, 100 µL of the isolate were spread onto MRS agar (Merck, Darmstadt, Germany). Subsequently, three antibiotic discs were placed on inoculated MRS agar and incubated at 37 °C for 24 h. After incubation, the diameter of the zone of inhibition around each disc was measured. The zone of inhibition represents the area where the growth of the microorganism was restrained, following the guidelines of the Clinical and Laboratory Standards Institute.

## 3. Results and Discussion

### 3.1. In Silico Genomic Insights of the Strain Lactiplantibacillus plantarum PU3

#### 3.1.1. Genome Overview

Nanopore sequencing offers several distinct advantages over traditional Next-Generation Sequencing (NGS) methods. One major benefit is its ability to generate long reads, enabling researchers to obtain more comprehensive and contiguous genomic information. This contrasts with short-read NGS technologies, which often struggle to resolve complex genomic regions and repetitive sequences. With Nanopore sequencing, assembling a bacterial genome becomes notably easier due to the longer reads provided by Oxford Nanopore Technologies (ONT). The long-read data allow for improved contiguity and reduce the need for extensive computational approaches to bridge gaps between short reads. Consequently, this streamlined assembly process helps researchers achieve more accurate and complete bacterial genome assemblies, enhancing our understanding of microbial diversity, evolution, and functional capabilities. Moreover, Nanopore MiniON devices are highly compact and easy to use, making sequencing technology accessible to every laboratory. It is important to note that ONT sequencing technology has witnessed significant advancements over the past years, where these improvements have resulted in enhanced read lengths, reduced error rates, and increased sequencing accuracy. Furthermore, the development of software-based calling algorithms has contributed significantly to the refinement of ONT sequencing data. All these factors will slowly but surely contribute to the independent use of ONT technology without the need for additional Illumina data [47].

Compared to other genomes of LAB species, the genomes of *L. plantarum* are larger, ranging from 2.91 to 3.70 Mb. This significant genome size might be associated with the bacterium’s capacity to thrive in various environmental habitats.

**Figure 1:**
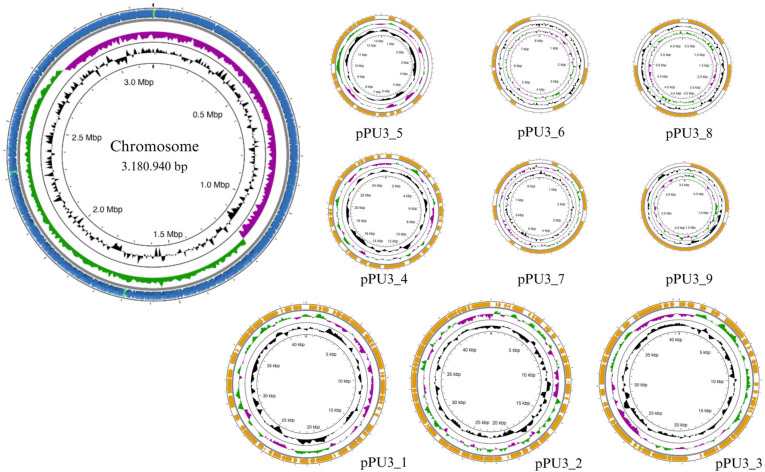
Circular map of the *L. plantarum* PU3 genome visualized using Proksee tool—bacterial chromosome and plasmids.

The complete genome of *L. plantarum* PU3 contains a single circular chromosome of 3,180,940 bp (coverage ×162) with a guanine-cytosine (GC) ratio of 44.65, with nine plasmids ranging from 44.900 bp to 3.512 bp with CG ratio of 35.22–41.08, respectively (Figure 1). The complete genomic sequences of *L. plantarum* PU3 have been submitted to the NCBI submission portal under accession numbers CP120642 and CP120643–CP120651 (plasmids). According to Capri et al. [9], the average full genome size and GC content of the *L. plantarum* strains are 3.32 Mb and 44.5%, respectively, with the number of plasmids ranging from 0 to 14, which complies with the results for the PU3 strain.

#### 3.1.2. Species Confirmation and Average Nucleotide Identity (ANI) of the Strain

**Figure 2:**
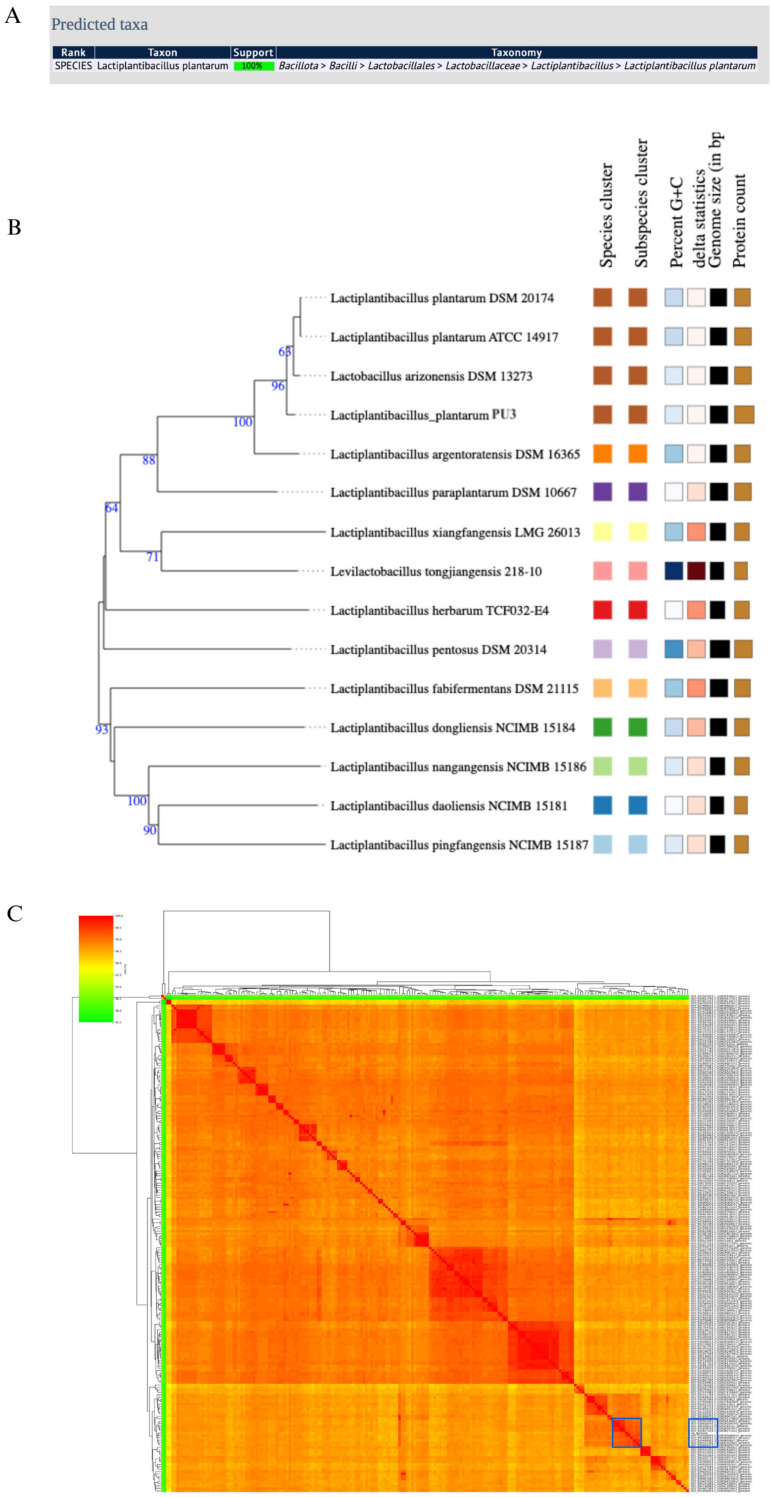
Species confirmation of the PU3 genome: (**A**) PubMLST results; (**B**) whole-genome phylogenetic tree generated by TYGS database; (**C**) ANI heatmap calculated between the PU3 genome and the complete full 207 *L. plantarum* genomes available in NCBI (the ten most similar genomes are marked with blue, and the ANI value ranged between 99.18–99.60%).

MultiLocus Sequence Typing (MLST) is a widely adopted method for the identification and typing of LAB [48]. It involves comparative sequence analysis of several housekeeping genes, resulting in a unique allelic profile of the microorganisms. MLST is considered a precise method, the results of which can be easily compared/exchanged between different studies [49]. In the case of the PU3 genome, PubMLST showed 100% support of the *L. plantarum* species (Figure 2B). Moreover, the genome similarity was evaluated by ANI and was calculated between the PU3 strain genome and the complete 207 *L. plantarum* genomes available in NCBI (Figure 2C). ANI values >95–96% were most often used as the criterion to confirm the species. The PU3 strain showed the highest ANI value of 99.60% with *L. plantarum* strain M19 isolated from raw milk motal cheese (GCA_018588605.2). Further, phylogenomic analysis using genome–genome comparisons in TYGS revealed that the PU3 strain is clustered with other representative strains in the database (Figure 2B). These results support that the PU3 strain belongs to *L. plantarum*. MOB-Typer results showed that the PU3 possessed four mobilizable, four non-mobilizable, and one conjugative plasmid (Table 1).

#### 3.1.3. Genome Annotation

**Figure 3:**
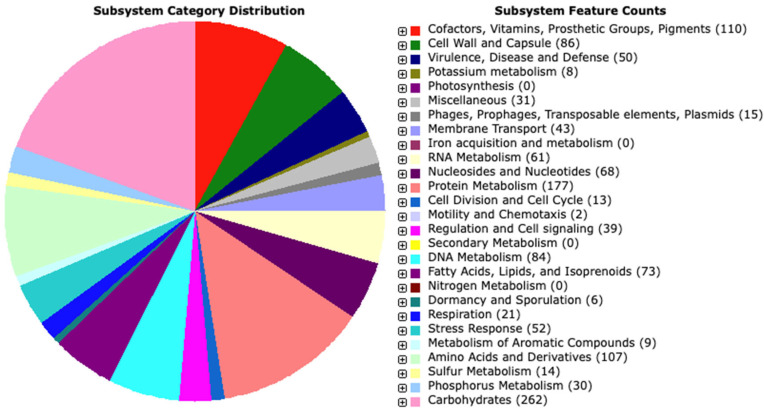
Distribution of *L. plantarum* PU3 subsystem gene functions. The pie chart shows the count of each subsystem feature and subsystem coverage.

We used the RAST server and enhanced the annotation with the BlastKOALA results. A total of 2962 genes, including 2874 protein-coding sequences (CDS), and 88 RNA genes (72 tRNAs and 16 rRNAs) were found. From the predicted CDS, 2335 genes (78.25%) were with annotated function, and 539 genes (21.74%) were hypothetical/unknown. RAST showed that proteins are involved in 257 subsystems (Figure 3). The detailed table with the RAST and BlastKOALA annotations is provided in Supplementary Table S1.

The distribution of various functional categories indicated the prevalence of genes associated with fundamental processes related to carbohydrates, amino acids, and their derivatives, as well as protein metabolism. Interestingly, the analysis revealed 110 genes involved in the production of cofactors, vitamins, prosthetic groups, and pigments. These genes notably participated in the biosynthesis of biotin, thiamin, pyridoxine, and folate, suggesting that strain PU3 possesses the capability to synthesize B vitamins—an advantageous characteristic for a potential probiotic strain. Furthermore, KEGG annotation by BlastKOALA assigned functional information to 1291 genes. Most notably, the protein families were associated with carbohydrate metabolism (162), signaling and cellular processes (154), environmental information processing (105), genetic information processing (138), amino acid metabolism (74), nucleotide metabolism (57), metabolism of cofactors and vitamins (47), etc.

#### 3.1.4. Carbohydrate-Active Enzyme (CAZyme) Annotation

The *L. plantarum* strains present plenty of diversity in the carbohydrate utilization-related key gene profiles. The utilization of carbohydrates by bacteria can be exploited to transform raw materials and producee valuable metabolites. CAZymes are sequence-based classified enzymes that can synthesize, modify, and disintegrate complex carbohydrates, which widely exist in LAB. The analysis of the PU3 genome in the dbCAN3 webserver using the predicted amino acid sequences as input revealed a total of 142 genes classified under five different CAZymes gene families as follows: 78 glycoside hydrolase (GH) genes, 45 glycosyltransferase (GT) genes, 1 carbohydrate esterase (CE) genes, 14 carbohydrate-binding modules (CBMs), and 2 auxiliary activity (AA) genes (Supplementary Table S3).

Bioinformatics analysis revealed that the PU3 strain has plenty of carbohydrate utilization genes. In that regard, the RAST tool reports genes related to Di- and oligosaccharides—Sucrose utilization (10), Maltose and Maltodextrin Utilization (22), Trehalose Uptake and Utilization (15), Beta-Glucoside Metabolism (35), Lactose and Galactose Uptake and Utilization (15), Lactose utilization (8), Lactate (15); Sugar alcohols—Glycerol and Glycerol-3-phosphate Uptake and Utilization (14); Polysaccharides—Alpha-Amylase (2); Monosaccharides—Mannose Metabolism (9), D-ribose utilization (5), D-gluconate and ketogluconates metabolism (5), D-Sorbitol (D-Glucitol) and L-Sorbose Utilization (8), Fructose utilization (10); and Aminosugars—Chitin and N-acetylglucosamine utilization (8). Nevertheless, the gene’s presence does not guarantee the substrate utilization by the strain; for that reason, we have undertaken further in vivo and in silico studies (see Section 3.2).

LAB, including *Lactobacillus* species, are capable of producing lactic acid, which constitutes more than 50% of the carbon from sugar as the primary end product during carbohydrate metabolism. This results in increased acidity in the fermentation broth, which effectively inhibits the growth of pathogenic bacteria in the gastrointestinal tract. In the PU3 genome, a total of five L-lactate dehydrogenase genes (P4B09_00555, P4B09_07360, P4B09_08060, P4B09_10620, and P4B09_15360) and three D-lactate dehydrogenase genes (P4B09_09595, P4B09_09600, and P4B09_14590) were identified.

#### 3.1.5. Identification of Probiotic Genes

Probiotic lactobacilli usually have a set of genes that encode proteins involved in stress responses (temperature, pH, bile, osmotic pressure, and oxidative stress) and are related to the adaptation of these organisms in the host gastrointestinal tract. In order to ascertain the probiotic properties of PU3 at the genomic level, we examined the genome for a variety of probiotic-related genes (stress resistance, bile salt hydrolase activity, adhesion, immunomodulatory activities, antioxidants, and vitamins). Based on published literature data [16,50,51,52,53,54,55,56], we identified 153 probiotic genes (some with multiple copies) related to different functions describing the probiotic genome capacity of the strain (Table 2). Since the table is quite extensive, a comprehensive description of the genes and Gene IDs within the PU3 genome is provided in Supplementary Table S2.

Genes encoding proteins involved in the gastrointestinal tract (GIT) stress response (acid and bile) were identified in the genome of *L. plantarum* PU3. These proteins include enolase *eno*, serine protease *HtrA*, ornithine decarboxylase, chaperones (*DnaK*, *DnaJ*, *groL/groEL*), Na^+^/H^+^ antiporter *NhaC*, F0-F1 ATP system genes, ATP-dependent *ClpP* protease, pyruvate kinase *pyk*, L-lactate dehydrogenases, Bile salt choloylglycine hydrolase, among others. The F0-F1 ATPase proton pump regulates cytoplasmic pH by pumping out H+ after ATP hydrolysis [57]. Cholylglycine hydrolase plays a crucial role in transforming conjugated bile acid into free bile acid, thus providing probiotic benefits within the gastrointestinal tract [58]. A total of 70 genes were identified that are associated with acid and bile tolerance. It is essential to acknowledge that acid tolerance can manifest through various mechanisms, such as alterations in the cell wall and biofilm development [59]. In this regard, biofilm formation serves as a means for survival and growth in challenging conditions, as it envelops viable bacteria within a protective polysaccharide capsule [59]. Genes associated with biofilm formation, like *luxS*, *comC*, *comD*, and *comE*, were also identified, and annotated.

The cell surface proteins of probiotic strains are responsible for their capacity to cling to host epithelium. The PU3 strain contains 19 genes coding for adhesion-related proteins, including lipoprotein signal peptidase II (*lspA*), elongation factor Tu (*tuf*), L-glyceraldehyde 3-phosphate reductase (*gpr*), sortase A (*srtA*), Fibronectin-binding protein *PrtF*, poly-beta-1,6-N-acetyl-D-glucosamine synthase *pgaC*, oligopeptide ABC transporter *OppA* and others, providing evidence of high adhesion capacity. The ability to bind to mucus has been associated with additional molecules, such as *Lactobacillus* surface protein A (*LspA*), which has been identified as a mucus-binding protein [60]. Moonlighting proteins, like elongation factor Tu and chaperonin *GroEL*, exhibit multiple functions, including adhesion to epithelial cells and/or extracellular matrix proteins, as well as host immunomodulation [61,62]. Similarly, α-enolase has been implicated in both adhesion to epithelial cells and/or extracellular matrix proteins, as well as interactions with plasma components [63,64].

The genome of the PU3 strain has several genes related to chitin and N-acetylglucosamine (NAG) utilization, including the chitin-binding proteins *cpb* (P4B09_13055, P4B09_15870), *nagE*, *nagB*, *nagA* and *nagR*. NAG and its derivatives have also been shown to support many aspects of immune health. Furthermore, studies report that *cpb* is able to bind directly to N-acetylglucosamine residues contained in chitin and in glycoproteins present on the surfaces of intestinal mucins and epithelial cells. Interestingly, one of the cpb proteins is plasmid-encoded (CP120643, P4B09_15870). These *cpb* proteins could thus perform important roles in the extracellular biology of *L. plantarum*, allowing adhesion to different surfaces present in different environments. The mucus-binding proteins present in PU3 might serve a twofold purpose: Firstly, participating in the attachment of this bacterium to the host cells, thereby enhancing the defense of the mucosal barrier and competitively excluding pathogens. Second, these proteins could also play a role in stimulating the mucin secretion by the host, similar to findings reported for other lactobacilli [64].

The antibacterial features can be categorized into two groups: protein and non-protein components. Among the protein substances, bacteriocins take precedence as antibacterial peptides that are biosynthesized by ribosomes. On the other hand, non-protein elements such as organic acids (lactic acid, citric acid, isobutyric acid, and acetic acid) are generated through lactobacillus fermentation. These organic acids have the capacity to decrease the pH level of the surrounding environment, resulting in the demise of pathogenic bacteria [65]. Additionally, exopolysaccharides from probiotic bacteria of *L. plantarum* also demonstrate antimicrobial properties [66,67].

The *Lactobacillus* genus possesses a distinctive characteristic, which is the production of exopolysaccharides (EPS). These EPS play a crucial role in various physiological functions, including stress tolerance, quorum sensing, and biofilm formation. Moreover, they find extensive applications in the food and pharmaceutical sectors. Typically, the EPS clusters consist of the *eps* genes, followed by several GT genes and a polysaccharide polymerase (genes P4B09_15175-P4B09_15110). In PU3, we have also observed such an EPS cluster having a lack of the *EpsA* gene, which is also observed for *L. plantarum* species by Deo et al. 2019 [68]. Furthermore, we observed another polysaccharide biosynthesis protein cluster consisting of five consequent genes P4B09_11025-P4B09_11045.

Probiotics can act as antioxidants to maintain redox balance in the gut. Twenty-five oxidative stress-related genes were found in *L. plantarum* PU3 encoding the whole thioredoxin system (*tpx*, *trxA*, *trxB*), glutathione (*gpx*, *gsr*, *gor*), and NADH (*ndh*, *npr*) antioxidant systems that are involved in ROS scavenging. Hydrogen peroxide and ROS can be degraded directly by NADH oxidase/peroxidase and catalase. These findings imply that *L. plantarum* PU3 may be resilient to various stressors and is congruent with the gastrointestinal tract’s adaptation traits. Additionally, adhesion-related protein helps the intestinal environment become effectively colonized and can eliminate undesirable gut microbes. Methionine sulfoxide reductase genes (*msrA*, *msrB*, and *msrC*) were shown to be able to repair proteins’ oxidized methionine residues caused by ROS [69], and were also identified in PU3. Earlier investigations have revealed that certain metabolites, such as exopolysaccharides and glutathione, synthesized by probiotics, possess the ability to mitigate oxidative damage, thereby providing preventive effects against aging and a range of chronic ailments [70]. These findings suggest that PU3 holds promise as a potential probiotic with antioxidant properties, promoting the reinforcement of intestinal epithelial barrier function and bolstering defense mechanisms against harmful pathogens.

Genes involved in temperature stress were also identified, which are important characteristics when the strain is used as a probiotic having resistance to stress conditions, prevalent during industrial processing and digestion. These include *CspA* (having four copies, one of which is plasmid-encoded in CP120647), members of the HSP20 family related to cold stress, heat shock protein *HtpX*, and heat-inducible transcriptional repressor *hrcA*, among others.

The LAB strains must adapt to osmotic changes that may hinder cell growth and metabolism during food processing. To accomplish this, they must have a molecular system against osmosis stress. These include genes from ABC transporters (*opuC*, *opuCA*, *opuBD*, and *opuA*) which also were identified. 

The criteria for designating bacterial strains as probiotics include several essential qualities. These strains must possess the ability to survive the journey through the gastrointestinal tract, adhere to and colonize intestinal epithelial cells, combat, and prevent the growth of harmful pathogens, enhance the existing microbiota, and regulate the immune system. Additionally, certain probiotics can synthesize important nutrients such as vitamins, particularly various B-group vitamins, and make them accessible to the host. In this regard, the *L. plantarum* PU3 has several genes that are related to the biosynthesis of enzyme CoA (8), foliate (15), pyridoxin (8), riboflavin (13), and biotin (5).

Human dietary consumption of fruits and vegetables, in addition to these products’ vitamins and dietary fiber content, is a source of polyphenol tannin. Tannins can form indigestible protein complexes and bind heavy metals. Tannins have also been associated with cancer [71]. *L. plantarum* is a LAB species that is most frequently encountered in the fermentation of plant materials where tannins are abundant. Tannase activity has been described in some *L. plantarum* strains [71,72,73]. PU3 also encodes tannase *tanB* (P4B09_02860), which can hydrolyze tannin into glucose and gallic acid, a harmful and anti-nutritional compound, which is further decarboxylated by *LpdD* (P4B09_06980) that encodes gallate decarboxylase activity.

#### 3.1.6. Antibiotic Resistance Gene (AMR) Prediction

Previous genomic analysis studies have shown that many Lactobacillus strains harbor AMR genes that mediate resistance to several antibiotics, including β-lactams, macrolides, chloramphenicol, and tetracycline [14,48,49,50]. Vancomycin resistance is intrinsic in *L. plantarum* strains, *vanY* and *vanX* genes encode D-Ala-D-Ala carboxypeptidase and D-Ala-D-Ala dipeptidase, respectively [32]. Thirteen genes related to antibiotic resistance were identified in the PU3 genome and were chromosome-encoded. These include resistance to Tetracycline—tetM, tetO (P4B09_06075); Macrolides—msr, vmlR (P4B09_06725); Chloramphenicol—catA (P4B09_13430); Beta-Lactams—Beta-lactamase C blC (P4B09_14440, P4B09_00710, P4B09_10960, P4B09_14440), penP (P4B09_00515, P4B09_00520, P4B09_07830), blA (P4B09_078250; Vancomycin—vanY (P4B09_10230), vanX (P4B09_09125); and Fluoroquinolones—gyrA (P4B09_05660), gyrB (P4B09_05665), parE (P4B09_13660).

#### 3.1.7. Virulence Factors and Bacteriocin-Encoding Genes

**Figure 4:**
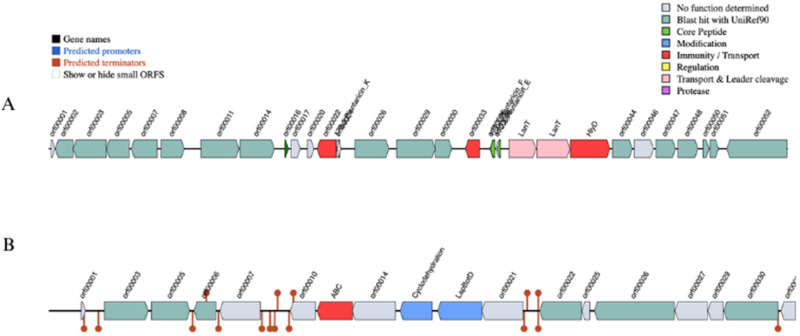
Genomic organization map of bacteriocins gene clusters in *L. plantarum* PU3 strain. (**A**) Plantaricin F class cluster (chromosome-encoded); (**B**) LAPs cluster (plasmid-encoded).

No virulence genes were detected by VFDB using the Abricate tool. However, the PU3 genome showed possession of bacteriocin class Plantacin F, with a cluster having Plantaricin E, F and K with chromosome location 1.561.101–1.586.810 bp (Figure 4A). These peptides usually inhibit both Gram-positive and Gram-negative bacteria. Furthermore, along core peptides, the bacteriocin cluster included four proteases (PlnT, PlnU, PlnV, and PlnW), bacteriocin immunity protein, plantaricin biosynthesis protein PlnY, response regulators PlnD, PlnI, and Bacteriocin ABC-transporter BlpB.

Additionally, both tools BAGEL4 and AntiMash tools predict linear azol(in)e-containing peptide cluster (LAPs) encoded within the plasmid CP120643 (14,366–36,554 nt) with primary genes: YcaO-like family protein (P4B09_15950) and SagB (P4B09_15955) (Figure 4B).

#### 3.1.8. CRISPR–Cas

CRISPR-CasFinder software identified a CRISPR array in the genome of the bacterium *L. plantarum* PU3, located on the chromosome 1.306.053–1.306.616 nt (evidence level 4). Close to the array, the software predicted the presence of a CAS-TypeIIA cas cluster that includes cas1, cas2, cas9, and csn2 (cas2_TypeI-II-III). These CRISPR-Cas systems are considered to help protect bacteria against foreign genetic elements such as phages, plasmids, and insertion sequences. They may also help prevent bacteria from acquiring resistance and virulence genes via horizontal gene transfer. Interestingly, recent studies showed that, among the 165 strains of *L. plantarum* evaluated, only 26 had CRISPR systems, and of those, 12 contained a type II system [74].

### 3.2. Characterization and Functional Analysis of the Strain L. plantarum PU3

#### 3.2.1. Assimilation of Various Carbon Sources by *L. plantarum* PU3

**Figure 5:**
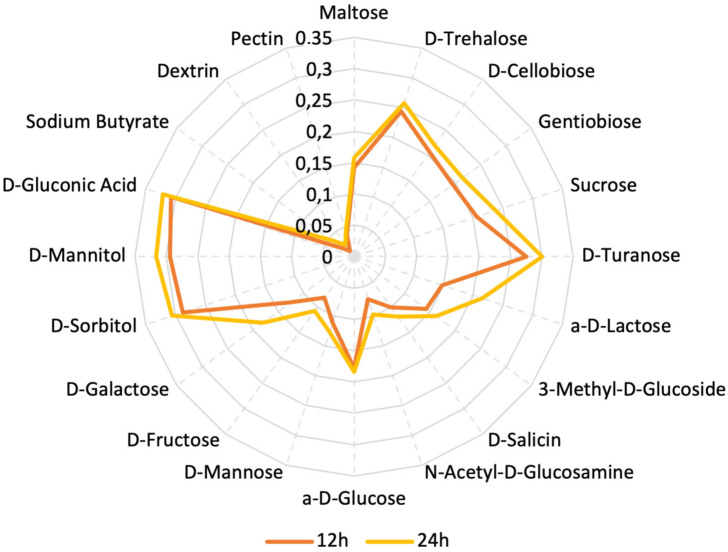
Assimilation of different types of carbon sources by *L. plantarum* PU3 expressed in optical density (OD).

We used the Biolog system to determine the utilization of various carbon sources, primarily carbohydrates. The plate used for analysis contained 31 different carbohydrates. After 24 h of incubation, we observed OD values indicating bacterial growth in the plate, corresponding to the ability to utilize the respective sugars. The carbohydrates we analyzed are Maltose, D-Trehalose, D-Cellobiose, Gentiobiose, Sucrose, D-Turanose, Stachyose, D-Raffinose, a-D-Lactose, D-Melibiose, 3-Methyl-D-Glucoside, D-Salicin, N-Acetyl-D-Glucosamine, N-Acetyl-D-Mannosamine, N-Acetyl-D-Galactosamine, a-D-Glucose, D-Mannose, D-Fructose, D-Galactose, 3-Methyl glucose, D-Fucose, L-Fucose, L-Rhamnose, Inosine, D-Sorbitol, D-Mannitol, L-Arabitol, myo-Inositol, Glycerol, D-Glucose-6-Phosphate, and D-Fructose-6-Phosphate. In Figure 5, the carbohydrates that strain was able to assimilate after being incubated on the plates are presented. Almost double the values of OD (optical density) were observed in the presence of gluconic acid, D-Mannitol, and D-Sorbitol when compared to the OD values of glucose. 

We did not observe any growth and consequently no change in the values of OD in the cells containing Stachyose, D-Raffinose, D-Melibiose, N-Acetyl-D-Mannosamine, N-Acetyl-D-Galactosamine, 3-Methyl glucose, D-Fucose, L-Fucose, L-Rhamnose, Inosine, L-Arabitol, myo-Inositol, Glycerol, D-Glucose-6-Phosphate, and D-Fructose-6-Phosphate.

Yang’s study [75] demonstrates that the bifunctional gene encoding aldehyde-alcohol dehydrogenase, *adhE*, is responsible for *L. plantarum*’s ability to utilize mannitol and sorbitol through cross-regulation by two DNA-binding regulators. In *L. plantarum* NF92, when *adhE* was examined, it was strongly induced, and the strain’s growth was suppressed when sorbitol or mannitol was used as a carbon source instead of glucose. This is a crucial characteristic for *L. plantarum*, enabling it to compete and survive in challenging environments where sorbitol or mannitol can serve as carbon sources. It is possible that *adhE* is strongly induced in the strain PU3, which may lead to an enchased ability to assimilate mannitol and sorbitol available in the environment; nevertheless, a further transcriptome investigation in that direction is needed.

**Figure 6:**
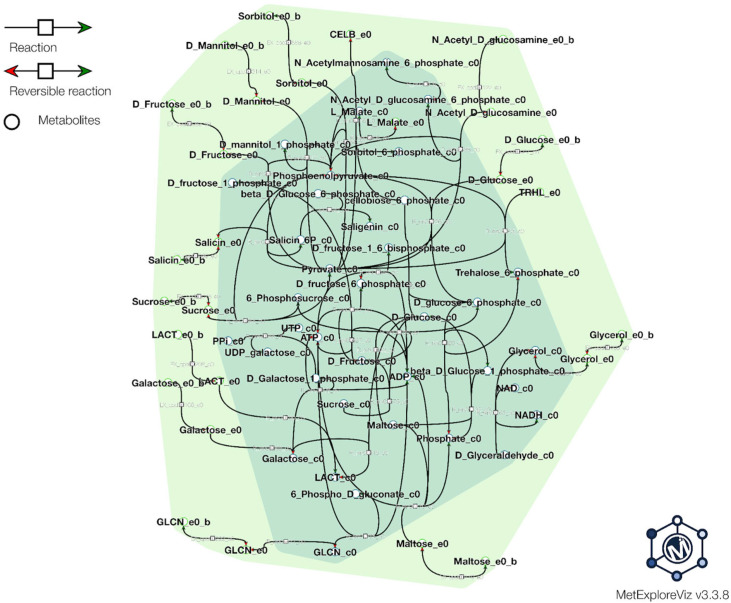
Carbohydrate source utilization simulated from iPU3 model. The figure visualizes several carbohydrate sources located in the extracellular compartment (e0) shown in light green, reactions how they enter the cytosol compartment (c0), and initial reactions and interlinks in the cell. (Not all reactions are shown due to visibility purposes; the visualization of the reactions is generated from MetExplorer tool).

**Figure 7:**
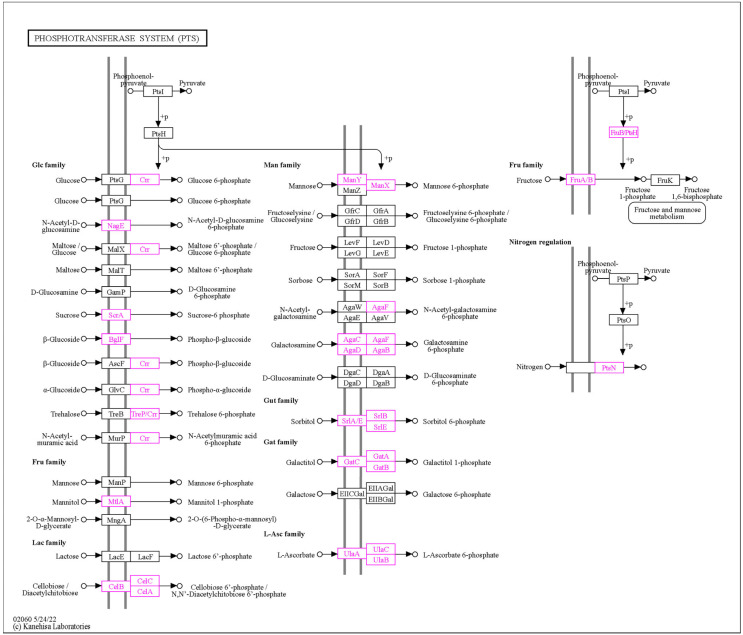
The sugar transporters of the phosphotransferase systems (PTS) in the genome of *L. plantarum* PU3 (showed in purple).

Genome-scale metabolic modeling (GSMM) is a powerful computational approach used to investigate the metabolic capabilities of organisms based on their genomic context and information. In the context of probiotic bacteria, GSMM has emerged as a valuable tool for understanding the functional potential of these beneficial microbes and also optimizing the process of probiotic production. We have used all available predicted and annotated proteins from the NCBI PGAP pipeline to feed into the ModelSEED database (https://modelseed.org/, accessed on 25 September 2023) for the generation of a draft model. The resulting model, iPU3, contained 595 genes and 1026 reactions. We have conducted flux balance analysis (FBA) in order to simulate the growth behavior on specific media with a single carbon source. The draft model showed the capability of generating a non-zero objective mass value and utilizing several carbohydrates as a single carbon source without preliminary gapfilling: Gluconic acid, Sorbitol, Sucrose, Trehelose, Cellobiose, Matose, Glucose, Fructose, Glycerol and Mannitol (Figure 6). Furthermore, due to limitations in carbohydrate enzyme annotation pipelines, causing mainly some transporters to be missed from draft reconstruction, gapfilling was used to enhance the model, and the additional carbohydrates also showed utilization, such as Lactose, Salicin and Galactose. Regarding the sugar transport system (phosphotransferase system, PTS), we found that the PU3 genome encoded 58 genes (some of which had multiple copies) for transporters that were predicted to be involved in the transport of various carbon sources (Supplementary Table S4) (Figure 7). The number of PTS transporters present in a species has been proposed to be due to the adaptation of species to their specific niches. Given the ecological flexibility exhibited by different *L. plantarum* strains, it is conceivable that they may require a substantial repertoire of PTS transporters to thrive in diverse environmental conditions. The metabolic model showed similar behavior and correspondence with the phenotype data observed in vitro. We provide the model for download as a Systems Biology Markup Language (SBML) file, as well as a tabular file with all available reactions and genes (Supplementary Data S2), which can be imported into various metabolic software for further exploration and improvement. The model can be further used to explore the metabolic capability, pathway and enzymatic repertoire of strain PU3 which can provide valuable knowledge for LABs’ functional properties as probiotics and to optimize the environment in the production process. 

In conclusion, the comprehensive integration of both wet and dry lab experiments provides evidence that *L. plantarum* PU3 has a strong capability in utilizing carbohydrates. This characteristic plays a crucial role in the colonization of the gastrointestinal tract and its probiotic effects. 

#### 3.2.2. Bile Salts and Acid Tolerance

**Figure 8:**
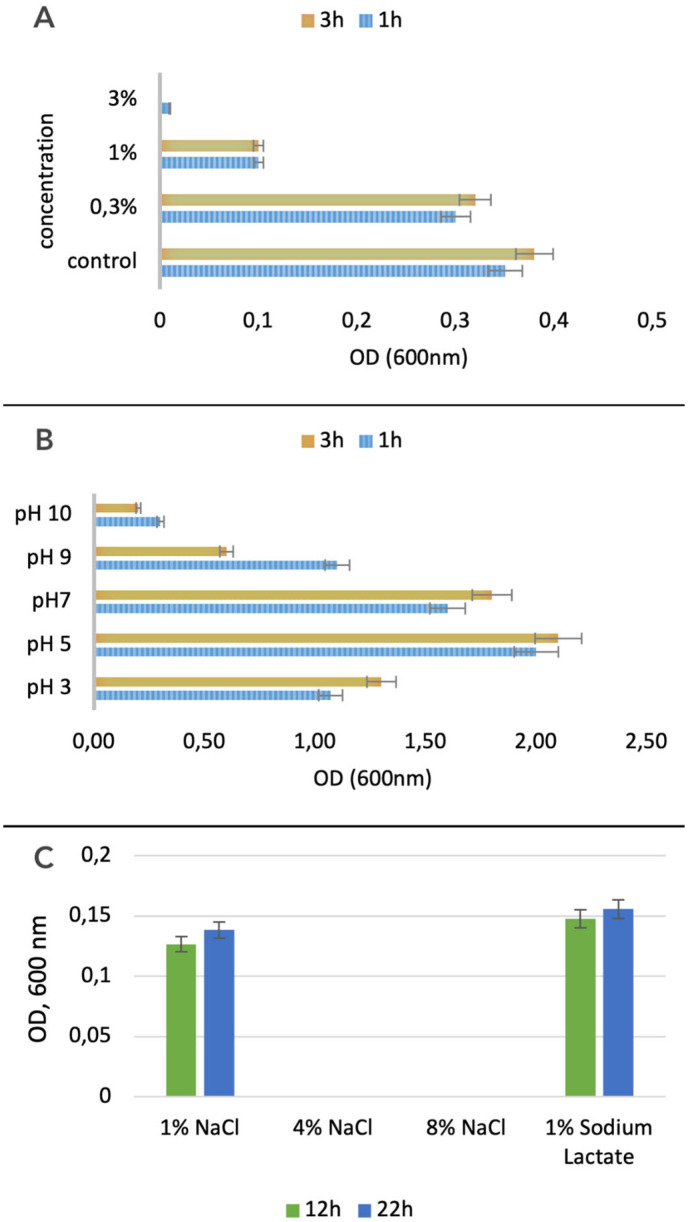
Bile (**A**), acidities (**B**) and osmotic (**C**) tolerance of *L. plantarum* PU3.

After culturing the *L. plantarum* PU3 strain in a medium containing various concentrations of bile salts, we observed growth in their presence. Despite the presence of a large set of genes involved in bile salt tolerance in PU3, the optimal growth was recorded at low concentrations of bile salts (0.3%), with almost no growth at a concentration of 3% (Figure 8A). Following their journey through the stomach, probiotic bacteria encounter the challenge of coping with bile juices within the intestine. A tolerance to bile concentrations ranging from 0.3% to 0.5% facilitates the probiotic’s successful establishment and colonization within the host’s gastrointestinal tract [76].

In addition to bile salts, we also examined the acid tolerance of the studied strain. We tested it in media with pH ranging from 3 to 10 and found the highest tolerance at pH 5. The *L. plantarum* PU3 has better tolerance at a pH in the acidic range compared to the alkaline one (Figure 8B).

Presently, commercially available lactobacilli possess specific health-enhancing characteristics [77]. For these bacteria to successfully reach the colon in a viable state during gastrointestinal transport, they must overcome significant challenges along the gastrointestinal tract, particularly the acidic environment of the stomach and the presence of bile salts in the upper sections of the small intestine. Most lactobacilli exhibit the ability to survive in highly acidic conditions with a pH as low as 2–3, thanks to their efficient metabolism of sugars, producing lactic acid [78]. However, there have been limited studies reporting diverse responses of lactobacilli to exposure to bile [79]. Apart from their typical physiological functions, bile acids act as mild acids with properties similar to detergents. They can be highly toxic to certain intestinal microbes, which naturally limit bacterial growth. In lactobacilli, the most prevalent strategies to counteract the harmful effects of bile salts include bile salt efflux, bile salt hydrolases, and alterations in the bacterial membrane composition [80]. An evaluation of 184 lactobacilli under gastrointestinal stress demonstrated that 12% of the strains showed a high growth capacity, while 38% did not grow after 24 h of cultivation at a bile salt concentration of 1.5% [81]. As a result, it is evident that there is considerable heterogeneity in the bile salt tolerance of lactobacilli.

#### 3.2.3. Osmotic Sensitivity of *L. plantarum* PU3

Through the Biolog system and the utilization of Gene III plates, we analyzed the osmotic durability of the *L. plantarum* PU3 strain by applying different concentrations of NaCl to the growth medium of the strain *L. plantarum* PU3. We reported the results at 12 h and 24 h from the start of inoculation on the Gene III plate. We recorded the optical density corresponding to bacterial growth. We also observed good tolerance to 1% sodium chloride and 1% sodium lactate. However, we did not observe any growth in the presence of 4% and 8% sodium chloride (Figure 8C).

Conventional probiotic-rich foods have traditionally been sourced from dairy products like yogurt and kefir. However, due to the growing popularity of dairy-free products and the rising demand for functional probiotic options, researchers have explored ways to introduce probiotics into various fruit- and vegetable-processing methods.

Non-dairy probiotic products, besides supplements, include fruit and vegetable drinks, fruit purees and pieces, jam and powders, chocolate, and even flan [82]. Recently, osmotically dehydrated fruits have been developed. In osmotic dehydration, pre-treated sliced fruits are immersed in an osmotic solution, for example, a sugar syrup (fruits) or a salt solution (vegetables), for a certain period of time at a temperature above room temperature and up to 60 °C. In this context, with the aim of using appropriate probiotic strains as additives in various products, osmotic tolerance is an important characteristic of the employed strain.

#### 3.2.4. Determination of Antibiotic Sensitivity of Strain *L. plantarum* PU3

We investigated the antibiotic sensitivity of *L. plantarum PU3* to eight antibiotics, divided into three groups according to their mode of action:

(1) Inhibitors of cell wall synthesis: Penicillin (10 µg), Amoxicillin (30 µg), III generation cephalosporins—Ceftriaxone (10 µg);
(2) Protein synthesis inhibitors: aminoglycosides—Amikacin (10 µg), Kanamycin (30 µg), Chloramphenicol (10 µg), macrolides-Erythromycin (15 µg);
(3) Inhibitors of nucleic acid synthesis: quinolones-Ciproflaoxacin (10 µg).

We found resistance of the examined strain to 4 out of 8 antibiotics used: Amikacin (10 µg), Ciproflaoxacin (10 µg), Erythromycin (15 µg), and Ceftriaxone (10 µg) (Table 3). 

Erythromycin macrolide antibiotic inhibits bacterial RNA-dependent protein synthesis by binding in the tunnel of the 50 S subunit in the ribosome, thereby blocking the extension of peptide synthesis. Amikacin inhibits bacterial RNA-dependent protein synthesis by binding to the tunnel of the 30 S subunit in the ribosome. According to ref. [83], Ceftriaxone is a third-generation cephalosporin that generally inhibits bacterial cell wall synthesis by acting on the cross-linking peptidoglycan. Cephalosporins are from the beta-lactams group. Beta-Lactamase is an enzyme found in drug-resistant bacteria involved in the hydrolysis of the β-lactam ring by substitution of an amino acid in the substrate (β-lactam antibiotic) [84]. The data obtained in vivo correspond to the results of the bioinformatic analysis, namely the presence of genes associated with antibiotic resistance (macrolides, fluoroquinolones, and beta-lactams).

The rise of antimicrobial resistance (AMR) is a worldwide concern, and probiotics are currently being examined closely for their potential role in contributing to AMR. In the past few years, there has been a growing trend of utilizing LAB as probiotics. These probiotic strains have gained popularity and are now widely available as health supplements and functional foods [85]. The presence of AMR genes in probiotic bacteria might enable them to function effectively in conditions where antibiotic use is prevalent, such as during infections treated with antibiotics. On the other hand, a concerning issue that has arisen is the potential transfer of genes from probiotic bacteria to other gut bacteria, including commensals and pathogens. This indicates a rising concern regarding the safety of probiotics [86,87,88]. While the coadministration of probiotics alongside antibiotics during antibiotic therapy has demonstrated health benefits, it also poses a potential risk of horizontal gene transfer of multidrug resistance to both pathogens and commensal microorganisms in the normal intestine [88]. This increase in antimicrobial resistance (AMR) has emerged as a significant problem in the healthcare field, leading to treatment failures and, ultimately, causing morbidity and mortality [89]. It is difficult to assume the potential of strain PU3 to participate in such a transfer despite the presence of 13 genes associated with resistance. Currently, the establishment of the gastrointestinal microbiome and resistome is primarily linked to the examination of fecal samples, neglecting in situ endoscopic investigations of the resistome and microbiome. Recent studies in this field report significant differences in the resistome between fecal samples and in situ. In recent studies, it has been demonstrated that a combination of probiotic strains from the commercial network can reduce the reservoir of antibiotic genes in healthy individuals who have not used antibiotics. In contrast, after specific antibiotic treatment, the same probiotic mixture enhances antibiotic-mediated resistome expansion. The probiotic influence on the resistome, particularly the strain PU3 we are exploring, should be the subject of further in-depth research, taking into account the influence of other strains presents in the gastrointestinal microbiome, and the concurrent use of antibiotics during the intake of a specific probiotic [86].

Bacteria can develop multidrug resistance through two main mechanisms: intrinsic resistance and acquired resistance. When probiotics are combined with antibiotics, various resistance mechanisms are developed, counteracting the bactericidal effects of the antibiotic. The specific resistance mechanisms may vary depending on factors such as the type of antibiotic used, the drug’s target site, the bacterial species involved, and whether the resistance is linked to a plasmid or chromosomal [90].

In the future, more in-depth research is needed on the capacity of a range of probiotics to participate in maintaining the reservoir of resistant genes in the human microbiome.

## 4. Conclusions

In conclusion, through the application of state-of-the-art third-generation Nanopore sequencing, *Lactiplantibacillus plantarum* PU3 unveiled its genomic intricacies, and a rich palette of 153 probiotic marker genes responsible for acid tolerance, bile resistance, adhesion, and responses to oxidative, osmotic and temperature stress. Moreover, our in vivo analyses provided evidence of the strain’s adeptness in utilizing diverse carbohydrates as growth substrates (with an affinity for D-sorbitol, D-mannitol, and D-Gluconic acid), a finding that was further corroborated through in silico examination of the associated metabolic pathways. Notably, the experimental validation of the strain’s acid, and osmotic and bile tolerance, suggest the PU3 strain’s adaptability and resilience within challenging environments.

The characteristics exhibited by the PU3 strain establish a promising foundation for exploring its full potential and contributions to the domain of probiotics. As such, further investigations are needed to fully unlock the strain’s capacities, paving the way for potential advancements in the field and also contributing to the existing body of knowledge concerning *Lactiplantibacillus plantarum.*

## Figures and Tables

**Table 1:** Plasmid characterization by MOB-typer tool.

Plasmid	Size	GC%	Relaxase_Type(S)	Predicted_Mobility	Nearest_Neighbor	Neighbor_Identification
PPU3_1	44900	39.00	MOBQ, MOBQ	conjugative	CP015967	*Lactobacillus plantarum*
PPU3_2	42197	39.94	MOBQ, MOBQ	mobilizable	CP035023	*Lactobacillus plantarum*
PPU3_3	40483	39.77	MOBQ	mobilizable	CP025284	*Lactobacillus plantarum* subsp. *plantarum*
PPU3_4	25867	41.09	MOBP, MOBQ	mobilizable	CP026509	*Lactobacillus plantarum*
PPU3_5	13241	39.27	-	non-mobilizable	CP010527	*Lactobacillus plantarum* subsp. *plantarum* P-8
PPU3_6	8689	35.93	-	non-mobilizable	CP005948	*Lactobacillus plantarum* subsp. *plantarum* P-8
PPU3_7	8053	35.23	-	non-mobilizable	KT149389	*Lactobacillus plantarum*
PPU3_8	6492	35.32	-	non-mobilizable	CP015127	*Lactobacillus plantarum*
PPU3_9	3512	37.30	MOBV	mobilizable	JX174167	*Lactobacillus plantarum*

**Table 2:** Probiotic gene markers identified in PU3 strain.

Probiotic Activity	Genes
Acid and bile tolerance	*rpsS*, *ppk*, *ackA_1*, *ackA_2*, *fabH_1*, *fabH_2*, *pgm*, *pepF*, *arcB*, *copA*, *dnaK*, *eno*, *pgk*, *dnaJ*, *groS/groES*, *grpE*, *htrA*
Acid stress	*atpA*, *atpB*, *atpC*, *atpD*, *atpE*, *atpF*, *atpG*, *atpH*, *clpB*, *clpP*, *dltC*, *gap*, *groL/groEL*, *ldh_1*, *ldh_2*, *ldh_3*, *ldh_4*, *pgi*, *plsC*, *pyk*, *recA*, *relA*, *tpiA*, *yjbM*, *ywaC*, *nhaC*, *lepA_1*, *lepA_2*,
Bile resistance	*argS*, *bsh_1*, *bsh_2*, *bsh_3*, *dps*, *glf_1*, *glf_2*, *glnA*, *nagB/gnp*, *oppA_1*, *oppA_2*, *oppA_3*, *oppA_4*, *pyrG*, *rplE*, *rplF*, *rpsC*, *rpsE*, *ppaC*, *cfa_1*, *cfa_2*, *cbh*, *celB*
Biofilm formation	*luxS*, *comC_1*, *comC_2*, *comD_1*, *comD_2*, *comE*
Adhesion	*exoA*, *lspA*, *PrtF*, *tuf*, *gpr*, *gapA*, *bgaB*, *epsA*, *epsB*, *pgaC_1*, *pgaC_2*, *pgaC_3*, *EpsC_1*, *EpsC_2*, *glnH*, *hsp33/hslO*, *srtA*, *dacC_1*, *dacC_2*, *cpb_1*, *cpb_2*, *EpsF_1*, *EpsF_2*
Oxidative stress	*fnr*, *nrdH*, *gpx*, *gsr_1*, *gsr_2*, *gsr_3*, *mntH_1*, *mntH_2*, *mntB*, *mntC*, *ndh_1*, *ndh_2*, *npr_1*, *npr_2*, *poxL_1*, *poxL_2*, *poxL_3*, *poxL_4*, *trxA*, *trxB*, *msrA_1*, *msrA_2*, *msrA_3*, *msrB*, *msrC*, *tpx*, *gor*
Temperature stress	*cspA_1*, *cspA_2*, *cspA_3*, *cspA_4*, *rnr*, *clpC*, *clpL*, *htpX*, *hrcA*, *hslV*, *HSP20*, *ctsR*
Immunomodulation	*dltD*, *dltC_1*, *dltC_2*, *dltB*
Ionic and heavy metal stress	*zntA*, *czcD*, *corA_1*, *corA_2*
Osmotic stress	*glpF_1*, *glpF_2*, *glpF_3*, *opuC*, *opuCA*, *opuBD_1*, *opuBD_2*, *opuA_1*, *opuA_2*

**Table 3:** Determination of antibiotic sensitivity of strain *L. plantarum* PU3.

Antibiotic Discs	*L. plantarum* PU3	
	CLSI ^a^	Mean ± Standard Deviation
AK (10 µg)	R	^b^
AMC (30 µg)	S	29.300 ± 0.500
CIP (10 µg)	R	^b^
C (10 µg)	S	25.364 ± 0.350
E (15 µg)	R	^b^
CRO (10 µg)	R	^b^
K (30 µg)	S	24.300 ± 0.150
P (10 µg)	S	20.435 ± 0.210

AK, Amikacin; AMC, Amoxicillin; AM, Ampicillin; C, Chloramphenicol; E, Erythromycin; CN, Gentamycin; K, Kanamycin; P, Penicillin; CIP, Ciproflaoxacin; CRO, Ceftriaxone; R, Resistant; S, Sensitive; CLSI, Clinical and Laboratory Standards Institute. ^a^ The inhibition zones are evaluated according to the standard values given by CLSI. Susceptible > 20, Intermediate = 15–19, Resistant ≤ 14 (CLSI, 2012) [42]. ^b^ Indicates no inhibition zone. Values are reported as mean ± SD of three separate replicates.

## Data Availability

Not applicable.

## Supplementary Materials

The following supporting information can be downloaded at https://www.mdpi.com/article/10.3390/microorganisms11102440/s1.
